# Cabozantinib and IL-27 combinatorial therapy for bone-metastatic prostate cancer

**DOI:** 10.3389/fmolb.2023.1259336

**Published:** 2023-09-28

**Authors:** Shreya Kumar, Grace E. Mulia, Marxa L. Figueiredo

**Affiliations:** Department of Basic Medical Sciences, College of Veterinary Medicine, Purdue University, West Lafayette, IN, United States

**Keywords:** prostate cancer, bone metastasis, immunotherapy, chemotherapy, interleukin-27, cabozantinib

## Abstract

**Introduction:** Prostate cancer is the second leading cause of cancer-related death among American men. Prostate tumor cells exhibit significant tropism for the bone and once metastasis occurs, survival rates fall significantly. Current treatment options are not curative and focus on symptom management. Immunotherapies are rapidly emerging as a possible therapeutic option for a variety of cancers including prostate cancer, however, variable patient response remains a concern. Chemotherapies, like cabozantinib, can have immune-priming effects which sensitize tumors to immunotherapies. Additionally, lower doses of chemotherapy can be used in this context which can reduce patient side effects. We hypothesized that a combination of chemotherapy (cabozantinib) and immunotherapy [Interleukin-27 (IL-27)] could be used to treat bone-metastatic prostate cancer and exert pro-osteogenic effects. IL-27 is a multi-functional cytokine, which promotes immune cell recruitment to tumors, while also promoting bone repair.

**Methods:** To test this hypothesis, *in vivo* experiments were performed where syngeneic C57BL/6J mice were implanted intratibially with TRAMP-C2ras-Luc cells that are able to form tumors in bone. Immunotherapy was administered in the form of intramuscular gene therapy, delivering plasmid DNA encoding a reporter gene (Lucia), and/or a therapeutic gene (IL-27). Sonoporation was used to aid gene delivery. Following immunotherapy, the animals received either cabozantinib or a vehicle control by oral gavage. Bioluminescence imaging was used to monitor tumor size over time.

**Results:** Combinatorial therapy inhibited tumor growth and improved survival. Further, RNA sequencing was used to investigate the mechanisms involved. Microcomputed tomography and differentiation assays indicated that the combination therapy improved bone quality by enhancing osteoblast differentiation and inhibiting osteoclast differentiation.

**Discussion:** Our conclusion is that a chemo-immunotherapy approach such as the one examined in this work has potential to emerge as a novel therapeutic strategy for treating bone-metastatic prostate cancer. This approach will enable a significant reduction in chemotherapy-associated toxicity, enhance sensitivity to immunotherapy, and improve bone quality.

## 1 Introduction

Bone is the most frequent metastatic site in patients with advanced prostate cancer (PCa). The presence of bone lesions contributes significantly to the decline in patient quality of life and increased mortality. These lesions result in weakened and brittle bones leading to fractures. Currently available treatments for such patients are mostly palliative and not curative. There is an urgent need for novel therapeutic strategies that can reduce the tumor burden and have additional therapeutic effects on the bone to undo the damage caused by the tumor. In the last few decades, combinational treatment strategies have emerged as a promising approach to treat various types of cancer. The use of multiple therapeutic strategies in combination can target the tumor using different mechanisms to reduce the occurrence of resistant cancer cells and improve efficacy. Additionally, therapeutics such as chemotherapies approved for other diseases can be repurposed in combination with other modalities such as immunotherapies to improve their anti-cancer potential.

An emerging chemotherapeutic to treat advanced PCa is cabozantinib (cabo), which is a tyrosine kinase inhibitor. It inhibits c-MET, vascular endothelial growth factor receptor (VEGFR-1, 2, 3), FLT3, c-KIT, and RET (Grüllich, 2014) along with AXL, all of which have been implicated in the process of tumorigenesis (Atkins and Tannir, 2018). In addition to their cytotoxic effects, chemotherapies can also have immunomodulatory effects, i.e., the treatment with certain drugs makes tumors more immunogenic (Zitvogel et al., 2023). Cabo can modulate the tumor microenvironment (TME) in multiple ways. It inhibits angiogenesis (by inhibiting VEGFR) and normalizes vascularization. This normalized tumor vasculature favors immune cell infiltration, making cabo an excellent partner for immunotherapies (Bergerot et al., 2019). Further, cabo has been shown to alter the phenotype of tumor cells by upregulating immune recognition markers and changing the immune-cell composition of the TME to a more anti-tumor repertoire. These changes have allowed cabo to be used in combination with a cancer vaccine to improve T cell mediated killing (Kwilas et al., 2014). More recently, cabo has been shown to induce immunogenic cell death *in vitro* in PCa cells (Scirocchi et al., 2021). All these studies point to the potent immunomodulatory effects of cabo that can be exploited to improve the efficacy of traditional or novel immunotherapies. Additionally, cabo also has been demonstrated to have pro-osteogenic effects by inducing a transient reduction in osteoclast (OC) number and increase in osteoblast (OB) numbers *in vivo* along with other modifications in bone structure and morphology (Haider et al., 2015).

Interleukin-27 (IL-27) is a versatile heterodimeric cytokine that has anti-tumor potential and has demonstrated limited toxicity in pre-clinical studies (Hisada et al., 2004). Many *in vivo* studies have demonstrated the anti-tumor effects of endogenous and exogenous IL-27. A study showed that IL27Rα-deficient mice had lower induction of cytotoxic T lymphocytes (CTLs) (Shinozaki et al., 2009). Natural Killer (NK) cells and Natural Killer T (NKT) cells have also been implicated in the anti-tumor activity of IL-27 (Wei et al., 2013). Such pre-clinical studies paved the way for using exogenous IL-27 as an anti-tumor immunotherapeutic. Our lab has extensively studied the effects of IL-27 on the bone microenvironment. Our studies have shown that IL-27 inhibits osteoclastogenesis and promotes OB differentiation, in prostate cancer *in vitro* models (Zolochevska et al., 2013a). Additionally, IL-27 was also able to improve bone density of mouse tibiae implanted with tumors. Some of the phenotypic changes caused in bone cells were due to the modulation of gene expression by IL-27 including the upregulation of pro-osteogenic genes in OBs, and downregulation of malignancy and osteoclastogenesis related genes in OCs (Zolochevska et al., 2013a). IL-27 also impacted osteoclastogenesis-related gene expression in T cells. As described above, bone metastasis is the leading contributor to PCa related morbidity and IL-27 is a promising therapeutic due to a combination of its anti-tumor and its pro-osteogenic effects. Further, IL-27 was modified at the C-terminus with a heptapeptide, LSLITRL (“pepL”), which targets the IL-6 receptor alpha subunit (IL-6Rα) (Su et al., 2005). Cancer cells have been shown to overexpress IL-6Rα and hence this receptor can be used to target tumor cells. One publication from our group (Figueiredo et al., 2020) demonstrated that the pepL could target a Gaussia Luciferase (Gluc) secreted protein to tumors following its gene delivery (pDNA-Gluc) and expression in skeletal muscle (relative to control non-specific C-terminal peptide, ns). Our group has also utilized the pepL to modify mouse IL-27 (IL-27pepL) and delivered this pDNA vector as a polyplex (complexed with cationic polymers) also into skeletal muscle *in vivo* using sonoporation. This work demonstrated the targeting ability of IL-27pepL relative to IL-27 containing a non-specific peptide (ns) at the C-terminus, as well as its improved bioactivity (Figueiredo et al., 2020). A major challenge in the field of cytokine therapy is the narrow therapeutic window due to the short half-life of cytokines (Donnelly et al., 2009). Gene therapy approaches prevent the need for repeated administration along with the flexibility to engineer the cytokine to enhance its bioactivity. Various non-viral gene delivery methods have been examined in the literature, with Sonoporation providing the advantage of organ or tissue-specific delivery of the gene since ultrasound waves can be applied to a localized region (Escoffre et al., 2013) such as skeletal muscle, as demonstrated in this study.

In the present study, a combination of IL-27pepL (referred to as IL-27 hereafter) and cabo was investigated in a bone-metastatic PCa model. It was hypothesized that the combination of IL-27 gene therapy and cabo could improve the immunogenicity of the tumor microenvironment, synergizing their anti-tumor effects while improving overall bone quality. A syngeneic mouse model was used to study the effect of IL-27, administered as gene therapy, and cabo, on intratibial tumors in an immune competent context. Tumor size was measured over time and tumors were harvested for analysis at the end of the study. Furthermore, the underlying molecular mechanisms involved in these anti-tumor effects were investigated, shedding light on the complex interplay between the tumor cells, bone microenvironment, and the immune response. Additionally, micro-computed tomography (µCT) was used to visualize and quantify bone changes resulting from the treatments.

RNA-sequencing showed that IL-27 gene delivered to mouse muscle modulated the immune-microenvironment of distal subcutaneous PCa tumors. *In vivo* experiments investigating the combination of IL-27 gene therapy combined with cabo demonstrated the potent inhibition of tumor growth by the combination compared to the monotherapy groups and the control group. Further, cabo administration increased the survival compared to the control group. The combination therapy had significant effects on bone quality as evidenced by microcomputed tomography (µCT) imaging. Morphometry analysis was used to determine the effect of the proposed therapy on bone parameters. *In vitro* differentiation assays were performed to elucidate the tumor-independent effects of IL-27 and cabo on bone cells including osteoblasts (OB) and osteoclasts (OC). The combination significantly promoted OB differentiation and inhibited OC differentiation.

Mechanistic evaluation of the therapeutic efficacy of IL-27 and cabo was performed by analyzing the differentially expressed genes (DEGs) obtained from sequencing total RNA from intratibial tumors. A number of immune-related pathways were enriched in the treatment groups compared to the control group. The immune infiltration in the intratibial tumors was estimated using TIMER2.0 analysis indicating an increase in pro-inflammatory immune cells and a decrease in regulatory immune cells. Finally, the efficacy of the treatment over time was evaluated by comparing DEGs in tumors harvested at two different time-points. Overall, this study proposes a novel combination therapy to treat bone-metastatic PCa which can reduce the tumor burden by enhancing the immunogenicity of the tumor microenvironment and improving bone quality by the restoration of the bone-remodeling homeostasis.

## 2 Materials and methods

### 2.1 Cell culture

Mouse adenocarcinoma cell line TC2Ras was cultured in Dulbecco’s modified Eagle’s medium–nutrient mixture F-12 (DMEM-F12) (Gibco, Waltham, Massachusetts) supplemented with 10% FBS (Hyclone, Cytiva, Marlborough, MA) and 1% antibiotic-antimycotic (Anti-Anti) (Gibco). TRAMP-C2 (ATCC, Manassas, VA) cells were transduced with a lentivirus expressing activated H-RasG12V (Lv-H-Ras) at a multiplicity of infection of 1 and a lentivirus expressing mouse androgen receptor at a multiplicity of infection of 1, resulting in TC2ras cells (Zolochevska et al., 2013b). TC2Ras cells were subsequently modified to express firefly luciferase (TC2Ras-Luc) to enable detection via bioluminescence imaging. The skeletal myoblast cell line C2C12 (ATCC) was cultured in Dulbecco’s Modified Eagle Medium (DMEM) (Gibco) supplemented with 10% FBS and 1% anti-anti. Pre-osteoblast MC3T3-E1 (clone 14) cells (ATCC) were cultured in α-MEM media (Gibco) supplemented with 10% heat inactivated FBS and 1% anti-anti. FBS was heat inactivated by incubation at 55°C for 30 min, then kept at 4 °C prior to use. RAW 264.7 cells (ATCC) were cultured in DMEM supplemented with 10% FBS and 1% anti-anti.

### 2.2 Vectors

The plasmid pCpGfree-Lucia (pCpG-Lucia) was purchased from Invivogen (San Diego, CA). To construct the pTSTA-ctrl vector, the components of the TSTA system (GAL4 and VP16) (Zhang et al., 2002) were cloned into pCpGfree-Lucia (VectorBuilder). A duplex encoding two Gly4Ser sequences as a linker (2xG4S) and the pepL sequence (LSLITRL) was synthesized by IDT (Integrated DNA Technologies, Coralville, IA, United States) to have 5′and 3′‘sticky ends’ compatible with XcmI and NheI, respectively. To construct the pTSTA-IL27pepL vector, this duplex was ligated into the pCpGfree-Lucia vector along with the TSTA elements (Supplementary Figure S1). Another pair of vectors was constructed with the pORF9 (plasmid open reading frame 9) backbone (Invivogen, San Diego, CA). The plasmid pORF9 was linearized with XcmI and NheI, and the duplex was ligated to create a final construct (pORF9-IL27pepL) encoding EBI3-p28-(2xG4S)-pepL. The ‘empty vector’ was called pORF9-0. All vectors were purified using GeneJET plasmid endotoxin free Megaprep kits (Thermo Fisher; Waltham, MA, United States) and subsequently precipitated and resuspended in nuclease-free water (Ultrapure DNAse/RNAse-free distilled water) for complexation.

### 2.3 *In vivo* experiments and bioluminescence imaging (BLI)

Male C57/BL6 mice (8–10 weeks of age) were obtained from Envigo. Mice were handled in accordance with an approved study protocol by the Purdue Animal Care and Use Committee (PACUC). TC2ras cells were prepared for implantation by adding trypsin (0.25% Trypsin-EDTA, Gibco, Billings, MT) to the cells and washing with sterile 1x DPBS. The pellet was resuspended in 1x DPBS at an appropriate concentration. For the intratibial model, 10^5^ TC2Ras-Luc cells were injected in each tibia in 10 μL sterile 1xDPBS. For the subcutaneous model, 3 × 10^5^ TC2Ras cells were injected in each flank in 50 μL sterile 1xDPBS. Implantation was performed under 2% inhaled isoflurane anesthesia delivered by high-flow vaporizer (VetFlo, Kent Scientific, Torrington, CT). The early treatment at day 1 post-tumor injection is to model the impact of microlesions or micrometastases, which still model aggressive tumor growth.

For gene delivery via sonoporation, the plasmids (pTSTA-ctrl or pTSTA-IL27pepL) were diluted to the concentration of 10 µg per muscle in a 20 µL volume. Sonovue microbubbles (Bracco, Milan, Italy) were added to the DNA just before injection (30% of the total volume). Following injection of the pDNA and microbubbles, ultrasound gel (Aquasonic 100, Fairfield, NJ) was applied to the shaved skin and the 6 mm probe of a KTAC4000 sonoporator (NepaGene, Japan) was used to apply ultrasound to the skin to stimulate the muscle below (1 MHz, 50% duty cycle, 2 W/cm^2^, 60 s).

Cabozantinib (LC Sciences, Houston, TX) was suspended in 15% polyvinylpyrrolidone (PVP). Cabo or PVP were administered (30 mg/kg, 100 µL) via oral gavage every day for 2 weeks post implantation and gene therapy. Tumor growth was monitored using bioluminescence imaging (BLI) following I.P. injection of luciferin substrate (Carbosynth, United Kingdom). Gene delivery was monitored by measuring the expression of Lucia in the muscle using BLI by intravenous injection of a different substrate with no cross-reaction with luciferin (Bhaumik and Gambhir, 2002), coelenterazine (MedChemExpress, Monmouth Junction, NJ). Images were acquired using a Spectral AMI Optical Imaging System (Spectral Instruments, Tucson, AZ).

### 2.4 Tissue collection

Mice were euthanized humanely using carbon dioxide from compressed cylinders according to the best practices determined by the American Veterinary Medical Association (AVMA) guidelines for the Euthanasia of Animals (2020), when animals presented signs of decreased quality of life or displayed any limb related discomfort. Blood was collected once a week from the submandibular vein and centrifuged to collect serum. In the subcutaneous model, tumors were harvested 1 week post implantation for RNA-sequencing analysis. In the intratibial model, 3 mice per group were euthanized 14 days post implantation, whereas the remainder of the mice (*n* = 4 mice) were allowed to progress to the ethical endpoint. Post-euthanasia, tumors were collected in RNAlater (Invitrogen, Waltham, MA) and preserved for further RNA isolation and analysis. Additionally, tibiae were collected in 10% buffered formalin (Fisher Scientific, Waltham, MA).

### 2.5 Microcomputed tomography (µCT) analysis of tibiae

Intact tibiae harvested from mice following fixation in 10% buffered formalin for 24 h and then stored in 70% ethanol were scanned using a Perkin Elmer Quantum GX µCT imaging system (PerkinElmer, Waltham, MA). Scans were performed with a 25 mm field of view, high resolution, and a scan time of 4 min. Scan parameters were: 90 kVp, 88 μA, and no frame averaging. Tibiae were reconstructed with an isotropic voxel resolution of 10 μm, and the images were analyzed using an adaptive thresholding algorithm in the manufacturer’s software (AccuCT 1.1 beta, PerkinElmer). The morphometric parameters measured included cortical area, total area, cortical thickness, bone volume, total volume, and trabecular bone surface area.

### 2.6 *In vitro* osteoblast/osteoclast differentiation

To collect conditioned media, C2C12 cells were seeded in a 6-well plate up to a confluency of 70%–80% in DMEM media. They were then transfected with either pORF9-0 or pORF9-IL27pepL plasmids using Lipofectamine 3000 (ThermoFisher, Waltham, MA) according to the manufacturer’s protocol. Briefly, 2.5 µg pDNA was complexed with 3.75 µL Lipofectamine 3000 reagent in the presence of 5 µL P3000 reagent in Opti-MEM media (Gibco). The complex was stabilized at room temperature for 15 min before being added to cells. 6 h later, transfection media was replaced with fresh DMEM supplemented with 2% FBS. Conditioned media (either control or containing IL-27pepL) were then collected after 24 h of incubation.

Osteoblast and osteoclast differentiation were assessed by differentiating MC3T3-E1 (clone 14, ATCC) preosteoblasts and RAW 264.7 monocytic cells (ATCC), respectively, in the presence of conditioned media and cabozantinib/DMSO. For osteoblasts, 5 × 10^4^ MC3T3-E1 cells were seeded in a 24-well plate and cultured with differentiation supplements (Millipore; ECM810, ascorbic acid (0.2 mM), 2-glycerol phosphate (10 mM), and melatonin (50 nM from day 10 onwards)) for 14 days. Conditioned media (with or without IL-27pepL) or cabozantinib (0.5 µM)/DMSO or a combination of IL-27pepL and cabozantinib was added to the cells to test the effect of the treatments on osteoblast differentiation. Cells were fixed with 10% buffered formalin and stained for alkaline phosphatase for 15 min in the dark (Millipore Sigma, Burlington, MA). The area occupied by positively stained cells (mature osteoblasts) was quantified using ImageJ.

For osteoclasts, 8 × 10^4^ RAW 264.7 cells were seeded in a 24-well plate and cultured with 50 ng/mL RANKL (R&D Systems, Minneapolis, MN) for 6 days. Similar to osteoblast differentiation, cells were treated with conditioned media (with or without IL-27pepL), cabozantinib/DMSO, or a combination. At the end of the differentiation period, 5 random fields per well were imaged using a microscope (20X). Cells fixed in 10% buffered formalin and were stained for Tartrate-Resistant Acid Phosphatase (TRAP) (Cosmo Bio, Japan). Multinucleated cells (>3 nuclei) that stained positive for TRAP were counted as differentiated osteoclasts.

### 2.7 RNA extraction

Tumors were preserved in RNAlater at 4°C for up to 1 week, following manufacturer’s protocols, to preserve RNA integrity for downstream applications. RNAlater was aspirated off tumor tissues and then kept in microcentrifuge tubes at −80°C. Total RNA was isolated from sections of these tumors. Briefly, 600 μL of RLT buffer + 10 μL of β-Mercaptoethanol were added to the tumor section, and homogenized with a PRO200 homogenizer (MidSci, ValleyPark, MO, United States) in three brief pulses of 10–15 s each at a mid-power. Lysates were then processed using a Qiagen RNAeasy kit (Qiagen) and eluted in 30 μL nuclease free water (Ultrapure DNAse/RNAse-free distilled water, ThermoFisher).

### 2.8 RNA-sequencing analysis

Total RNA was sequenced by LC Sciences (Houston, TX) with analysis support. Before sequencing, RNA integrity was checked with an Agilent 2100 Bioanalyzer to ensure a minimum RNA Integrity Number (RIN) of 8. Illumina’s TruSeq-stranded-mRNA protocol was followed to prepare a poly (A) RNA sequencing library and Poly (A) tail-containing mRNAs were purified using oligo- (dT) magnetic beads. The purified poly (A) RNA was fragmented using divalent cation buffer in elevated temperature. Agilent Technologies 2100 Bioanalyzer High Sensitivity DNA Chip was used to perform quality control analysis and to quantify the sequencing library. Illumina’s NovaSeq 6000 sequencing system was used to perform paired-ended sequencing. Transcript reads that contained adaptor contamination, low quality bases and undetermined bases were removed using cutadapt (Martin, 2011) and perl scripts in house. FastQC (http://www.bioinformatics.babraham.ac.uk/projects/fastqc/) was used to verify sequence quality, and HISAT2 (Kim et al., 2015) was used to map reads to the *Mus musculus* genome.

StringTie (Pertea et al., 2015) was used to assemble reads in the transcript, perl scripts and gffcompare were employed to merge the transcriptomes and reconstruct a comprehensive transcriptome. StringTie and ballgown (http://www.bioconductor.org/packages/release/bioc/html/ballgown.html) were used to estimate transcript expression levels. The mRNA expression levels were calculated using StringTie by determining the Fragments Per Kilobase of transcript per Million mapped reads (FPKM). Outliers within treatment groups were identified using Pearson correlation analysis, considering an outlier to be any value with an R value less than 0.9. Differential expression analysis of mRNAs between two groups was performed using the R package DESeq2 (Love et al., 2014) between two different groups (and by R package edgeR (Robinson et al., 2010) between two samples). The mRNAs with *p*-value below 0.05 and absolute fold change ≥ 1.5 were considered differentially expressed mRNAs. Metascape (Zhou et al., 2019) was used for ontology assessment to identify potential cellular processes and pathways associated with the observed differential gene expression.

### 2.9 Immune cell profiling using RNA-sequencing data

To predict the immune-composition of the tumor microenvironment, TIMER2.0 was used (Shalapour et al., 2015). TIMER2.0 uses the immunedeconv package (Sturm et al., 2019) to estimate immune cell abundance using six algorithms taking tissue-type specific information into account (PRAD, prostate adenocarcinoma, for example,) in order to improve the estimation accuracy. This tool provides a detailed immune cell profiling (>100 cell types), using recently characterized algorithms (including EPIC, quanTIseq and TIMER) in this comprehensive platform.

### 2.10 Statistics

Statistical analysis of RNA-sequencing data was performed via R package DESeq2. For other analyses, GraphPad Prism version 6 for Windows (GraphPad Software, San Diego, CA) was used to calculate statistically significant differences among groups, using unpaired *t*-test or ANOVA with *p*-value <0.05 considered significant. *TumGrowth* (https://github.com/kroemerlab) was used to calculate significance and overall survival for the *in vivo* tumor growth experiment.

## 3 Results

### 3.1 IL-27 gene therapy promotes immunomodulation within the tumor microenvironment

The overall impact of IL-27 gene therapy on the tumor microenvironment was assessed by initially implanting subcutaneous TC2Ras tumors in mice followed by the intramuscular delivery of plasmid expressing IL-27pepL or a control plasmid, aided by sonoporation. The tumors were harvested 7 days post-implantation and total RNA was isolated for sequencing.

**FIGURE 1:**
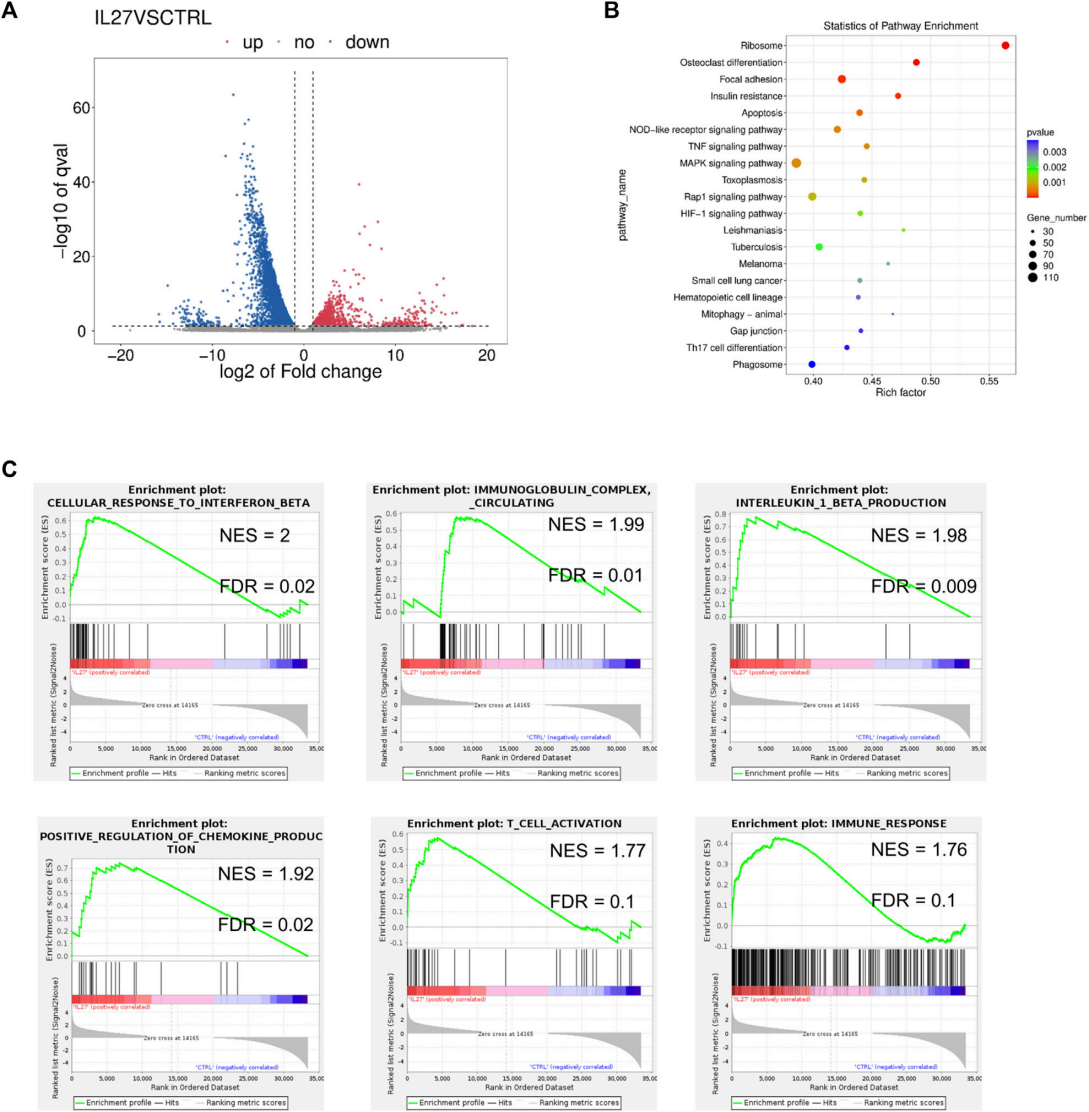
RNAseq analysis of IL-27 treated tumors. **(A)** Volcano plot representing the DEGs in IL-27 treated tumor compared to empty vector treated tumor, **(B)** GSEA pathway enrichment (KEGG) scatter plot showing the number of genes involved in the enriched pathways, **(C)** Enrichment plots obtained from GSEA showing the normalized enrichment score (NES) and the false discovery rate (FDR) of some select pathways in the GO database.

A total of 1620 differentially expressed genes (DEGs) were upregulated and 3724 DEGs were downregulated in the IL-27 treated tumor compared to the control vector-treated tumor (Figure 1A). STAT1 (fold change = 2.53) and STAT4 (fold change = 4.22) were significantly upregulated in IL-27 treated tumors (Figure 1A). IL-27 exerts many of its anti-tumor effects via STAT1 mediated signaling, indicating that the delivered IL-27 is bioactive (Figueiredo et al., 2020). In PCa, IL-27/STAT1 has been shown to decrease angiogenesis and increase the production of immunomodulatory chemokines such as CXCL10 and CXCL11 (Shen and Lentsch, 2004). In this experiment, IL-27 treatment significantly upregulated CXCL11 with a fold change of 20.59.

GSEA analysis was used to detect enriched pathways associated with the DEGs. The top enriched pathways in the Kyoto Encyclopedia of Genes and Genomes (KEGG) database included *OC differentiation* along with immune-associated pathways such as *TNF signaling*, and *Th17 differentiation* (Figure 1B). IL-27 also enriched the processes of *IFNβ response*, *immunoglobulin response*, *IL-1β production*, and *chemokine production* in the Gene Ontology (GO) database (Figure 1C). Additionally, *T cell activation* and other immune-related responses were enriched. Additionally, DEG lists were examined with Enrichr (Xie et al., 2021), and the pathways enriched in Reactome 2022 were obtained (Table 1). The top pathways (lowest *p*-values) included immune-related processes involving *cytokine signaling*, *interleukin signaling*, *adaptive immune-response*, *innate immune system*, and *pro-inflammatory cell recruitment*. Recently, it was discovered that type I IFN signaling is suppressed in bone metastatic prostate tumor cells and this mechanism could be driving metastasis by reducing immunogenicity (Owen et al., 2020). Restoration of IFN signaling could improve the efficacy of immunomodulatory therapeutics. These data confirmed that IL-27 gene therapy is able to modulate the tumor immune-microenvironment and can potentially facilitate the efficacy of other immunomodulatory and cytotoxic therapeutics such as cabozantinib.

**TABLE 1:** Top 10 enriched pathways following IL-27 gene therapy.

Enriched pathways in Reactome 2022 (upregulated DEGs)	*p*-value
Immune System R-HSA-168256	5.31E-16
Cytokine Signaling In Immune System R-HSA-1280215	2.16E-09
Interleukin-10 Signaling R-HSA-6783783	3.15E-08
Signaling By Interleukins R-HSA-449147	5.23E-08
Striated Muscle Contraction R-HSA-390522	9.29E-08
Adaptive Immune System R-HSA-1280218	2.83E-07
Interleukin-4 And Interleukin-13 Signaling R-HSA-6785807	3.73E-06
Muscle Contraction R-HSA-397014	7.26E-06
Toll-like Receptor Cascades R-HSA-168898	1.54E-05
Innate Immune System R-HSA-168249	3.60E-05

### 3.2 The combination of cabozantinib and IL-27 inhibits the growth of PCa in the bone and improves survival

**FIGURE 2:**
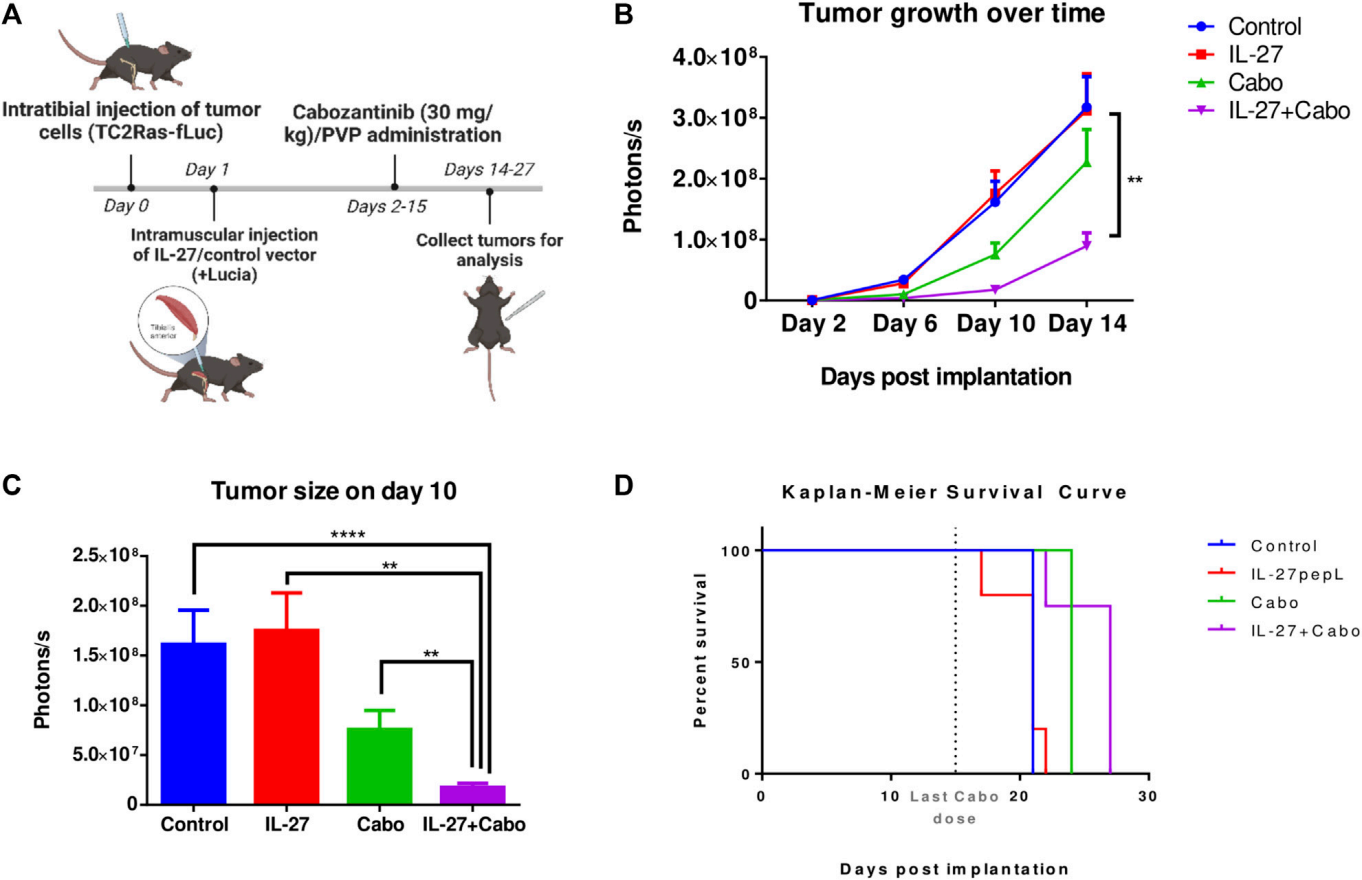
*In vivo* evaluation of IL-27 and cabo efficacy against syngeneic intratibial prostate tumors. **(A)** Experiment methodology and **(B)** tumor growth over time measured by BLI, *n* = 7 mice per group, mean ± SEM (Type II ANOVA with Tukey’s multiple comparison test, Control vs. IL-27+cabo **p < 0.01) **(C)** Tumor sizes compared on day 10, *n* = 7 mice per group. Mean ± SEM (Wilcoxon Rank-Sum test with Holm adjustment, ****p < 0.0001, **p < 0.01). **(D)** Survival curve, cabo (*p* = 0.0355) and cabo + IL-27 (*p* = 0.0063) treatments provided significant survival advantage over the control group, *n* = 4 mice per group, *p*-value calculated using the Cox regression model with Bonferroni adjustment. Analysis performed using TumGrowth (https://github.com/kroemerlab) to calculate significance.

To investigate the therapeutic potential of IL-27 and cabo on bone-metastatic PCa, a syngeneic intratibial mouse tumor model (n = 7 mice per group) was used. TC2Ras-Luc cells were injected intratibially promote tumor development. The TSTA IL-27pepL-Lucia gene therapy was delivered to the tibialis anterior muscle using sonoporation in half the cohort. A control vector containing only Lucia was used in the other half of the cohort (Figure 2A). Additionally, cabo or polyvinylpyrolidone (PVP, vehicle control) was administered via oral gavage for 2 weeks. During the course of the experiment, tumor growth was measured by BLI (Figures 2B, C). Randomly selected animals were euthanized on day 14 (*n* = 3 per group) while the rest of the mice continued to stay in the study until endpoint for survival study (Figure 2D). Day 14 was selected as the time-point for mechanistic investigation of therapy efficacy based on previous pilot experiments (data not shown). Secreted Lucia was detected in the serum 14 days post implantation indicating successful transfection of the muscle cells (Supplementary Figure S2).

BLI showed that the combination of cabo and IL-27 inhibited prostate tumor growth in the bone (Figures 2B, C). The inhibitory effects of the combination were significantly greater than the monotherapies, potentially indicating a synergistic effect. While cabo monotherapy inhibited tumor growth and extended survival, IL-27 monotherapy did not demonstrate these therapeutic effects. However, in combination, the therapeutic effects were significantly greater.

### 3.3 Cabozantinib and IL-27 treatment improved bone quality *in vivo*

**FIGURE 3:**
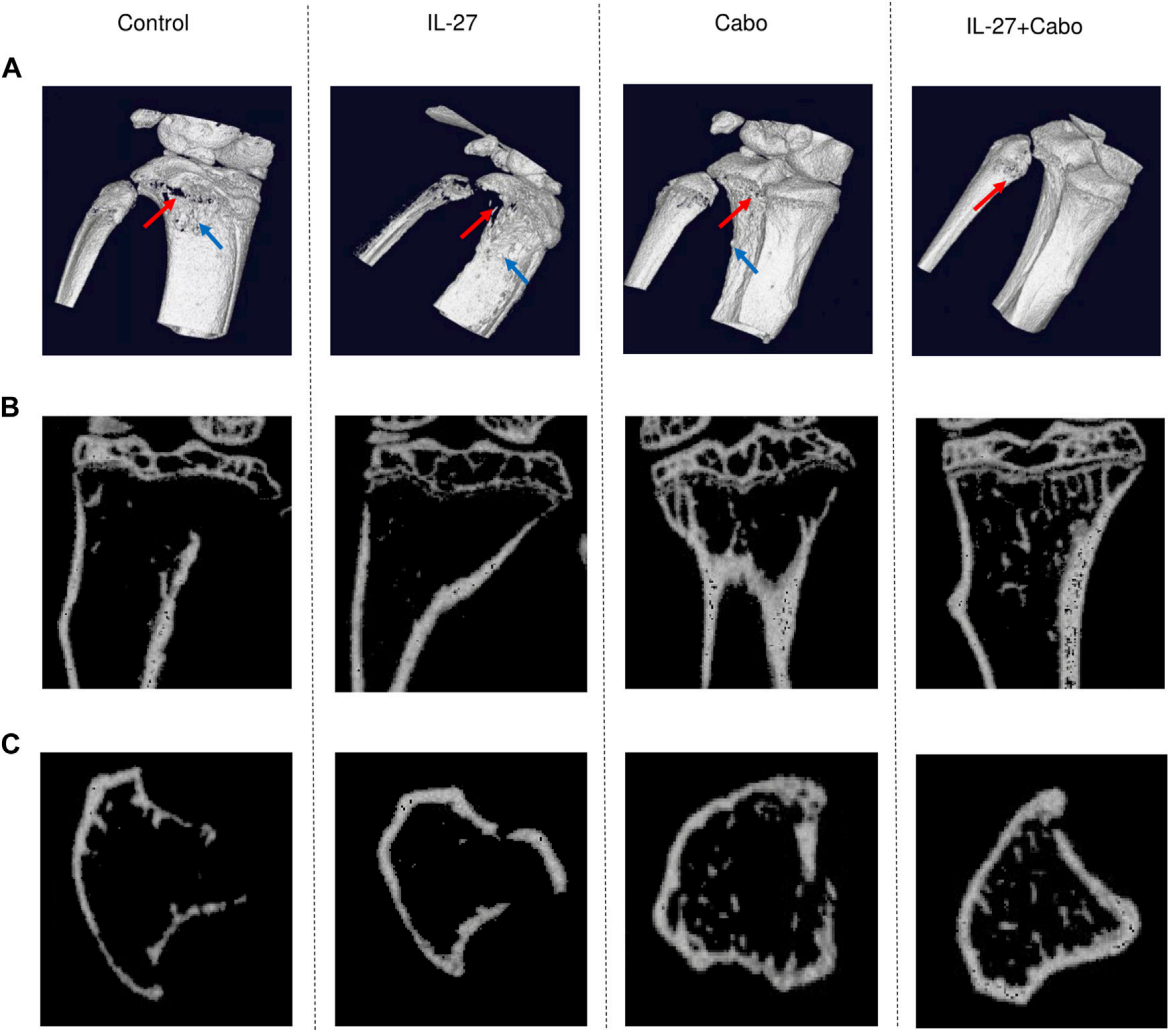
Micro-tomographic (µCT) analysis of tibiae from tumor-bearing mice treated with IL-27, cabo, and combination (IL-27+cabo). 14 days post intratibial TC2Ras tumor implantation, tibiae were harvested from mice and imaged using µCT. **(A)** Representative 3D reconstruction (10 µm) of the tibiae showing tumor-induced osteolysis (red arrows) and osteoblastic lesions (blue arrow). Representative 2D **(B)** lateral, and **(C)** cross-sectional µCT images showing the retention of bone structure in treated animals relative to control.

To assess the impact of the proposed treatment combination on the bone microenvironment, µCT analysis was performed using tibiae harvested from mice implanted with intratibial TC2Ras tumors and treated with either IL-27/cabo monotherapy or the combination (IL-27+cabo). 3D reconstruction of the bones showed that the tumors caused significant damage to the tibiae, resulting in mixed osteolytic and osteoblastic lesions. Control animals showed severe degradation of the bone structure compared to IL-27/cabo treated animals, with the combination group having the best bone quality (Figure 3). The 2D lateral and cross-sectional images further demonstrated the improvement in bone quality due to IL-27 and cabo (Figure 3B, C). Moreover, the IL-27+cabo group had a trabecular architecture closer to normal relative to each of the individual treatments and the control (vehicle/control vector) group (Figure 3C).

**FIGURE 4:**
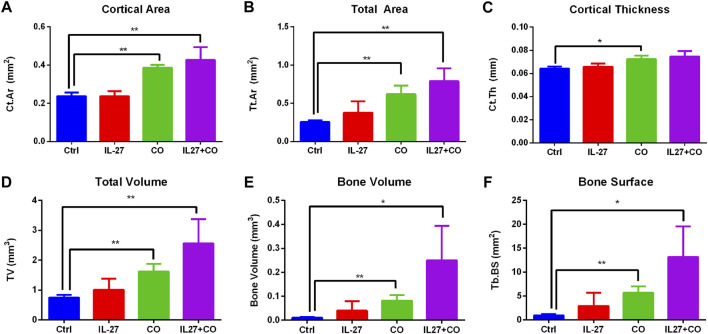
Morphometric analysis of bone parameters. AccuCT software was used to quantify bone quality and structure. Overall parameters such as **(A)** bone volume **(B)** total volume **(C)** total area, trabecular parameters such as **(D)** trabecular bone surface area, and cortical parameters such as **(E)** cortical area and **(F)** cortical thickness were measured. Cabo and IL-27+cabo groups showed significant improvements in bone parameters relative to the control group treated with vehicle (PVP) and control vector. Data presented as mean ± SEM, *n* = 6 bones, Mann-Whitney test, *p < 0.05, **p < 0.01.

Next, morphometry analysis was performed to quantify bone-related parameters. A 100 slice-thick region of interest (ROI) located 1 mm from the growth plate was selected. The ROI size was kept constant for all scans. Cabo treatment resulted in a significantly increased total bone area (cortical and trabecular) (Figures 3A, B). The cabo and IL-27+cabo groups showed significant improvements in bone volume and total volume relative to control (Figures 3D, E). Although the effect of the IL-27 monotherapy did not reach statistical significance in these data, a trend in improved bone quality was observed with the IL-27 treatment increasing bone volume and trabecular bone surface area. Confirming the trends observed in the cross-sectional images, trabecular bone surface and cortical parameters such as cortical area and thickness also improved upon cabo/combination therapy compared to control (Figures 3A, C, F). In summary, cabo treated groups displayed significantly improved bone quality in tumor-bearing mice through an enhanced retention of trabecular structure and cortical thickness relative to controls. Figure 4.

### 3.4 IL-27 and cabo treatment enhances osteoblast differentiation and inhibits osteoclast differentiation *in vitro*

IL-27 and cabo can improve bone quality in tumor-bearing mice by directly reducing tumor burden; additionally, it was found that IL-27 and cabo have direct activity on bone cells. To test the effects of IL-27 and cabo combination on bone cells, *in vitro* experiments were performed to model our *in vivo* experimental setup (i.e., genes delivered to muscle). First, conditioned media (CM) was generated by transfecting C2C12 muscle cells with either a control vector (pTSTA-ctrl) or an IL-27pepL vector (pTSTA-IL27). Next, CM was collected at 24 h and 48 h intervals and was used to culture bone cells *in vitro* undergoing differentiation in the presence of supplements.

**FIGURE 5:**
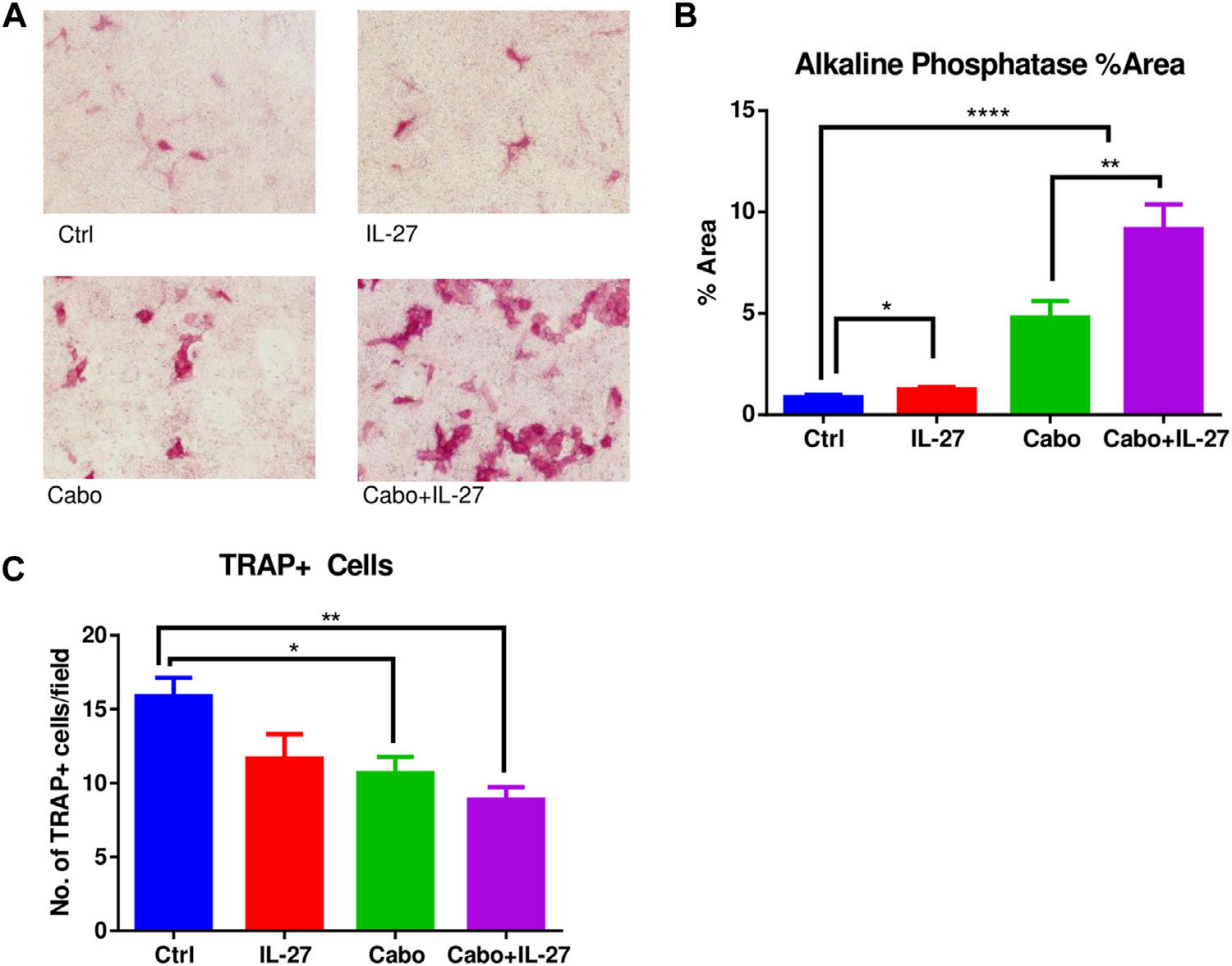
The impact of IL-27 and cabo on osteoblast and osteoclast differentiation. **(A)** Representative images showing alkaline phosphatase positive cells after 14 days of differentiation of MC3T3-E1 (clone 14) cells in the presence of DMSO (Ctrl), conditioned media (CM) containing IL-27, cabo, or combination of cabo + IL-27 CM in the presence of differentiation supplements, **(B)** ImageJ was used to quantify the area occupied by alkaline phosphatase positive cells, 5 random fields of 3 wells per group were imaged, **(C)** RAW264.7 cells were cultured with CM containing DMSO (ctrl), IL-27, cabo, and cabo + IL-27 ± RANKL for 6 days followed by staining for TRAP + cells. Multi-nucleated cells were counted in 5 random fields of 3 wells per group. Data presented as mean ± SEM, 5 random fields were imaged per well of the experiment conducted in triplicate, One-way ANOVA with Tukey’s multiple comparison test, *p < 0.05, **p < 0.01, ****p < 0.0001.

3.5 MC3T3-E1 (pre-osteoblastic, clone 14) cells were cultured either with CM (ctrl or IL-27) and/or cabo (0.5 µM)/DMSO vehicle in the presence of OB differentiation supplements for 14 days. Alkaline phosphatase staining showed that treatment with IL-27 CM in combination with cabo significantly increased OB differentiation relative to monotherapy with IL-27 or cabo (Figures 5A, B). Next, RAW264.7 cells (monocyte/macrophage cell line) were similarly cultured with CM (ctrl or IL-27) and in the presence of or absence of osteoclastogenic stimulus RANKL (50 ng/mL). On the sixth day, the cells were stained for TRAP activity and multi-nucleated (>3 nuclei) TRAP + cells were counted as a measure of OC differentiation (Figure 5C). Both cabo and the combination (cabo + IL-27) groups showed significantly fewer TRAP + cells relative to the control or IL-27 monotherapy group. These results indicate that, in addition to their anti-tumor effects, IL-27 and cabo improve bone quality by influencing both OB and OC differentiation.

### 3.5 Differential expression of genes in tumors

**FIGURE 6:**
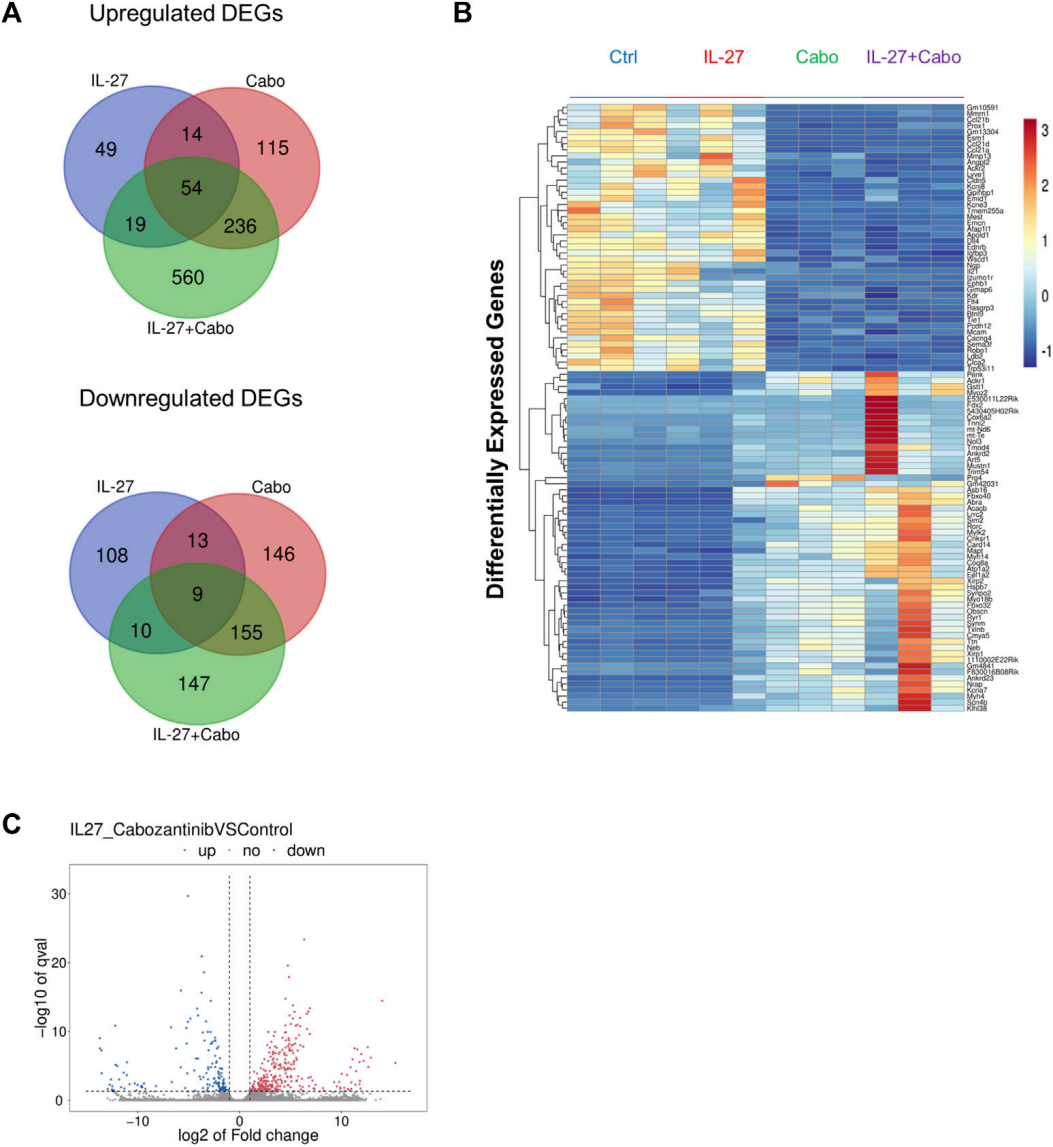
Summary of DEGs: **(A)** Summary of overlap in genes upregulated and downregulated across treatment groups using Venn diagrams made using the Van de Peer Lab Venn diagram tool (http://bioinformatics.psb.ugent.be/webtools/Venn/). Genes were considered differentially expressed if Log2(FC) ≥ 1.5 or ≤ −1.5 and *p*-value < 0.05 as determined by DESeq2 analysis, **(B)** Heatmap of DEGs in tumors treated with IL-27, cabo, and IL-27+cabo compared to control, *n* = 3 tumors per group **(C)** volcano plot showing DEGs in the combination group (IL-27+cabo) vs. control.

Considering the significant anti-tumor and pro-osteogenic effects of IL-27 and cabo combination therapy, RNA-sequencing was used to further elucidate the mechanisms contributing to the therapeutic effects of these agents utilizing an *in vivo* intratibial TC2Ras model. Tumors were harvested 14 days post implantation from all groups (control, IL-27, cabo, and IL-27+cabo) and total RNA (*n* = 3 tumors per group) was sequenced and analyzed as described in the materials and methods section. The Differentially Expressed Genes (DEGs) were identified across different treatment groups (Log2 (FC) ≥ 1.5 or ≤ −1.5 and *p* < 0.05) relative to the control tumor samples (Figure 6).

The unique DEGs downregulated by IL-27+cabo therapy were analyzed using *Enrichr* (Xie et al., 2021)*.* The enriched pathways [according to the Reactome 2022 database (Gillespie et al., 2022)] were obtained (Table 2). These downregulated DEGs were involved in T cell related processes including *PD-1 signaling, CD4 downregulation,* CD28 dependent pathways such as *Vav1 Pathway*, *PI3K/Akt Signaling,* and *co-stimulation,* indicating a possible role of T cell subsets in the therapeutic activity of IL-27+cabo*.* It has been shown that IL-27 gene therapy can deplete T_reg_ population and inhibit IL-2 signaling (Zhu et al., 2018). A similar response was observed here, with *Interleukin-2 Family Signaling* being one of the key enriched pathways obtained from the downregulated DEGs. Another enriched pathway was *RUNX3 Regulates Immune Response* and *Cell Migration,* which suggests that the combination treatment was able to improve anti-tumor immunity by reducing the population of immunosuppressive cell types since RUNX3 is associated with the regulation of inflammatory cells and the generation of T_reg_ cells in the tumor microenvironment (Li et al., 2012; Manandhar and Lee, 2018). As expected, *FLT-3 signaling*, which is a cabo target (Yakes et al., 2011), was also downregulated.

**TABLE 2:** Enriched pathways (unique DEGs) downregulated due to IL-27+cabo therapy.

Enriched pathways in Reactome 2022 (downregulated DEGs)	*p*-value
Nef-mediated Downmodulation Of Cell Surface Receptors By Recruitment To Clathrin Adapters R-HSA-164938	4.05E-04
PD-1 Signaling R-HSA-389948	4.69E-04
Binding And Entry Of HIV Virion R-HSA-173107	7.89E-04
Role Of Nef In HIV-1 Replication And Disease Pathogenesis R-HSA-164952	1.00E-03
Nef Mediated CD4 Down-regulation R-HSA-167590	0.001459
Costimulation By CD28 Family R-HSA-388841	0.001584
Generation Of Second Messenger Molecules R-HSA-202433	0.00165
CD28 Dependent Vav1 Pathway R-HSA-389359	0.003374
Early Phase Of HIV Life Cycle R-HSA-162594	0.006018
Translocation Of ZAP-70 To Immunological Synapse R-HSA-202430	0.006788
Phosphorylation Of CD3 And TCR Zeta Chains R-HSA-202427	0.009347
CD28 Dependent PI3K/Akt Signaling R-HSA-389357	0.012269
Metal Ion SLC Transporters R-HSA-425410	0.015536
CD28 Co-Stimulation R-HSA-389356	0.025814
Signaling To P38 Via RIT And RIN R-HSA-187706	0.036217
Interleukin-2 Family Signaling R-HSA-451927	0.041513
FLT3 Signaling Thru SRC Family Kinases R-HSA-9706374	0.043303
WNT Mediated Activation Of DVL R-HSA-201688	0.043303
RUNX3 Regulates Immune Response And Cell Migration R-HSA-8949275	0.043303
PI5P, PP2A And IER3 Regulate PI3K/AKT Signaling R-HSA-6811558	0.043385

**FIGURE 7:**
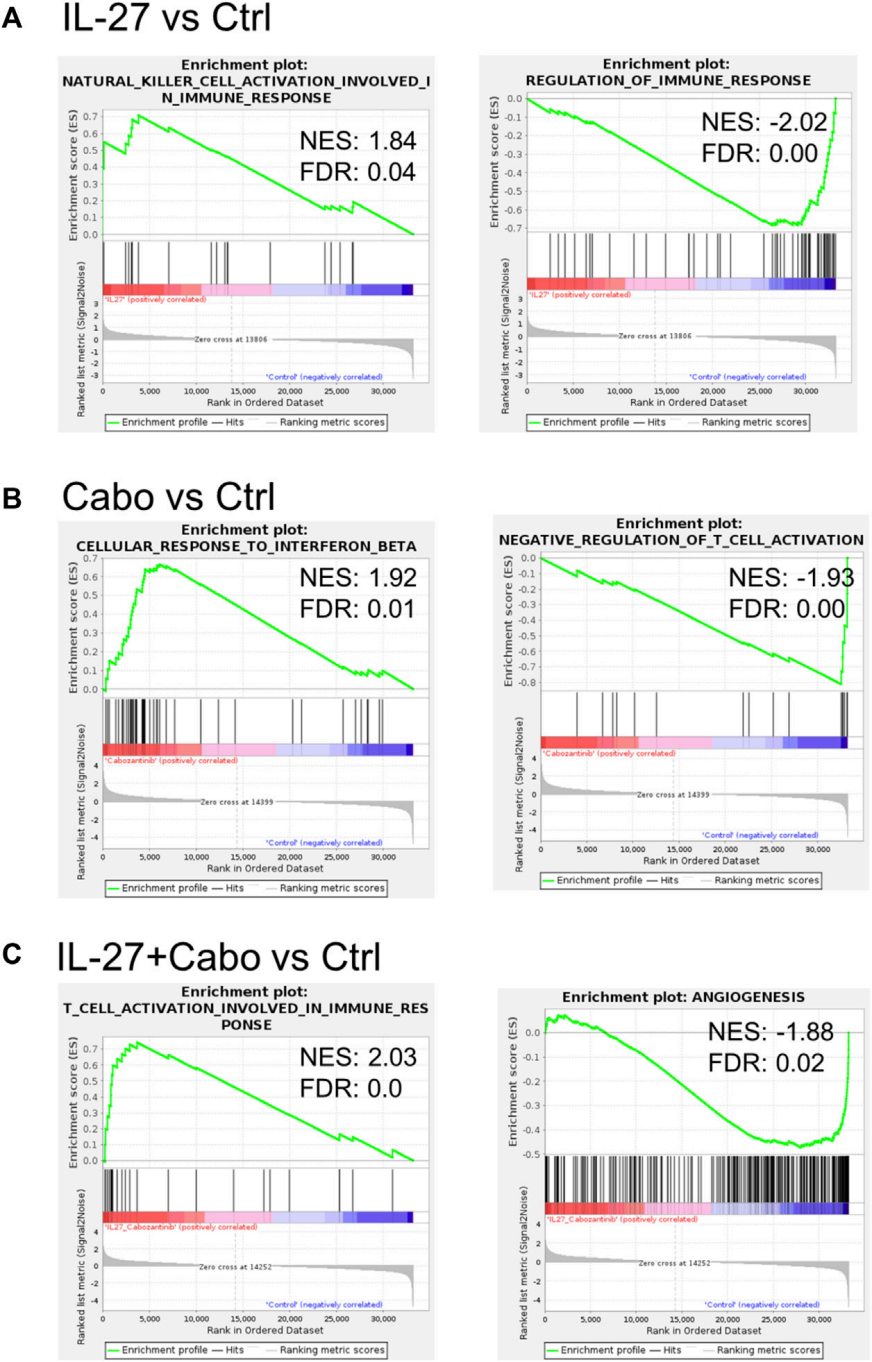
IL-27 and cabo treatment enhance immune activity in the TME: Select GSEA analysis for expressed genes ranked by fold change of expression **(A)** IL-27 vs. ctrl, **(B)** cabo vs. ctrl, and **(C)** IL-27+cabo vs. ctrl against the biological process as indicated. IL-27 monotherapy improved NL cell activity and reduced immune regulation. Cabo improved response to IFNβ and reduced the regulation of T cell activation. The combination of both therapies synergistically improved T cell activation and inhibited angiogenesis.

A Gene Set Enrichment Analysis (GSEA) of the DEGs showed that in IL-27 treated tumors, 45 gene sets were enriched at false discovery rate (FDR) < 25%. Gene Ontology (GO) enrichment revealed that 22 biological processes, 4 cellular components, and 11 molecular functions were enriched. GSEA results showed that IL-27 treatment is positively associated with *NK cell activation* (Table 3) and negatively associated with *B cell signaling*, and *regulation of immune response* (Figure 7A). In cabo-treated tumors, 151 gene sets were enriched at FDR>25%. GO enrichment showed that 428 biological process, 86 cellular component, and 150 molecular function terms were impacted by cabo. Cabo treatment was positively associated with immune-related pathways such as *cellular response to IFNβ*, *positive regulation of chemokine production*, (Table 3) and negatively associated with *negative regulation of T cell activation*.

**TABLE 3:** Immune-related GO pathways enriched by treatment groups vs. control.

Treatment group	Enriched GO pathways	NES	FDR q-val
IL-27	NK cell activation involved in immune response	1.841485	0.049932
cabo	Cellular Response to IFNβ	1.929386	0.007146
cabo	Positive regulation of chemokine production	1.840582	0.027069
IL-27+cabo	Type I IFN receptor binding	2.200875	0
IL-27+cabo	Cytokine receptor binding	2.075944	0.001023
IL-27+cabo	NK cell activation involved in immune response	2.050994	0.001352
IL-27+cabo	T cell activation involved in immune response	2.033695	0.001579

The combination of IL-27 and cabo promoted the best anti-tumor response and the highest survival rates in mice. In these tumors, 110 gene sets were enriched at FDR<25%. GO enrichment indicated that 421 biological process, 69 cellular component, and 114 molecular function terms were impacted by the combination therapy. The GO enriched terms indicated a combination of both IL-27 and cabo’s effects (Table 3). For instance, similar to IL-27 treated tumors, *NK-cell activation* was associated with IL-27+cabo. Cabo contributed to the efficacy of the combination by increasing type I IFN response and decreasing tyrosine kinase activity. Interestingly, the combination treated tumors had DEGs associated with *T cell activation*, a function not found to be enriched in the individual therapy groups, suggesting a synergistic response. Overall, the combination of IL-27 and cabo can immunomodulate the tumor microenvironment in the bone by activating anti-tumor immune cells and suppressing anti-inflammatory responses.

### 3.6 Estimation of immune infiltration in the intratibial tumors

GSEA analysis of DEGs showed that IL-27 and cabo therapy can modulate the immune-microenvironment *in vivo* and potentially alter the immune-cell infiltration compared to control tumors. A web-based server, TIMER2.0, was utilized to estimate the differences in immune-infiltration among the different groups based on DEGs obtained from RNA-sequencing (Li et al., 2020). This server combines six state-of-the-art established algorithms to infer immune-cell composition in the tumors based on bulk transcriptomic profiles.

**FIGURE 8:**
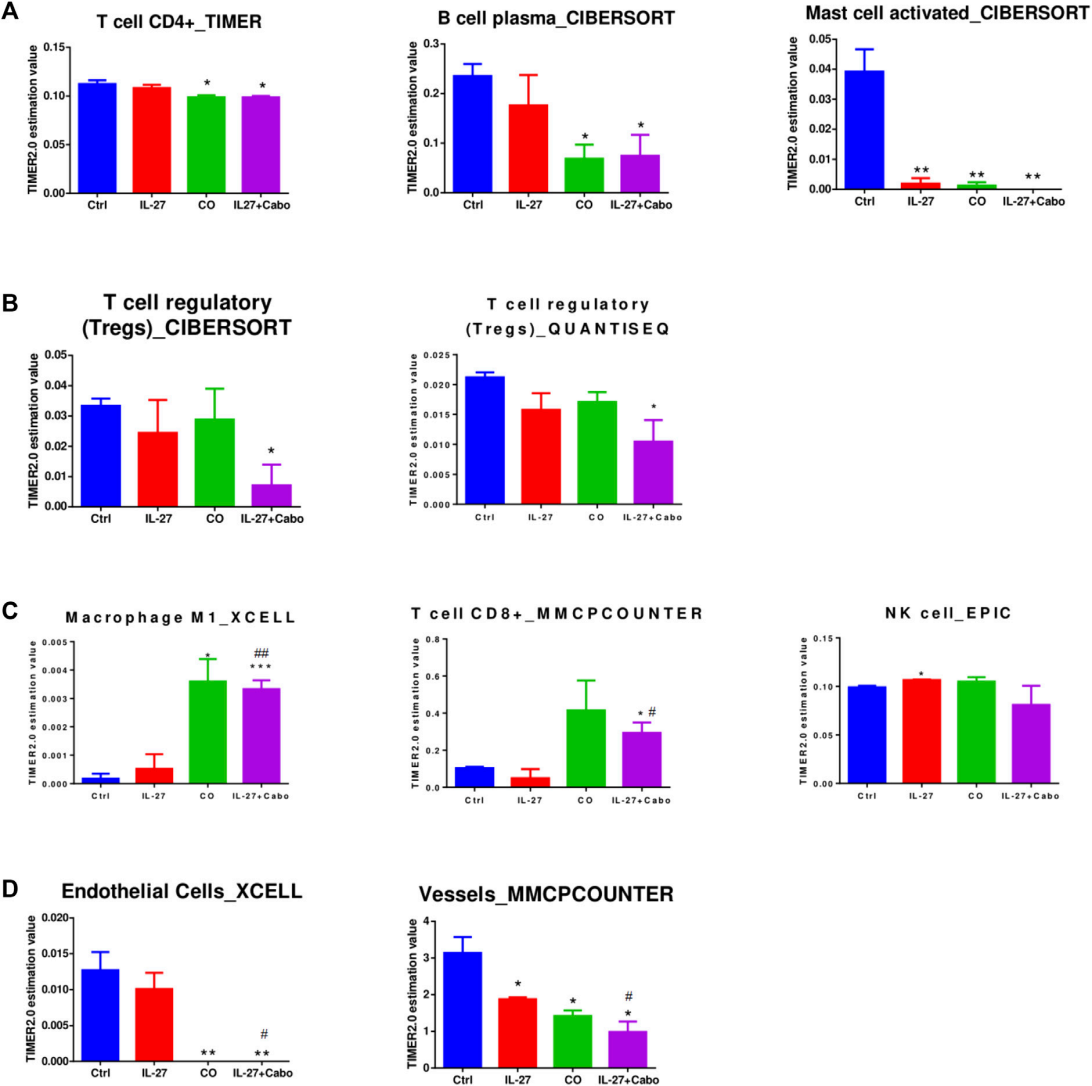
Assessment of Immune Cell Infiltration in Treated Tumors. To evaluate immune cell infiltration, we analyzed differentially expressed genes (DEGs) derived from RNA-sequencing data using the TIMER2.0 algorithms. The data is presented as the mean abundance estimation value ± SEM (*n* = 3 RNA samples per group). Significance levels are indicated as follows: *p < 0.05, **p < 0.01, ***p < 0.001, determined by unpaired *t*-test compared to the control (ctrl); #*p* < 0.05, ##*p* < 0.01, indicating significance levels compared to the IL-27 group, also determined by unpaired *t*-test.

Tumors treated with cabo showed a significant decrease in CD4^+^ cells and B-plasma cells compared to control tumors (Figure 8A). Depending on their subtype, B cells can play pro- or anti-tumorigenic roles. Certain immunosuppressive plasma cells expressing IL-10 and PD-L1 can impede immunogenic oxaliplatin chemotherapy in PCa (Shalapour et al., 2015). This could be one of the mechanisms by which tumors become resistant to chemotherapy. All the treatment groups were predicted to have decreased activated mast cell levels. Mast cells can encourage the progression of PCa by promoting epithelial to mesenchymal transition (EMT) (Ma et al., 2018). Suppression of mast cells by IL-27/cabo can potentially inhibit metastatic tumor growth.

In concordance with the GSEA analysis, T_regs_ are predicted to be downregulated in tumors treated with the combination of IL-27 and cabo (but not the monotherapies), indicating a potentially synergistic effect through immune-regulatory mechanisms (Figure 8B). The combination-treated tumors were also predicted to have increased infiltration of inflammatory/tumoricidal M1 macrophages as well as CD8+T cells that could contribute to a robust anti-tumor response in these tumors (Figure 8C). The treated tumors were predicted to have a lower abundance of endothelial cells/vessels, which could be due to reported anti-angiogenic effects of IL-27 (Shimizu et al., 2006) and cabo (Yakes et al., 2011) (Figure 8D). Overall, IL-27 and cabo work together to increase the infiltration of anti-tumor immune cell types, decrease the presence of immune-suppressive regulatory immune cells, and inhibit angiogenesis in the tumor.

### 3.7 Efficacy of treatment over time

**FIGURE 9:**
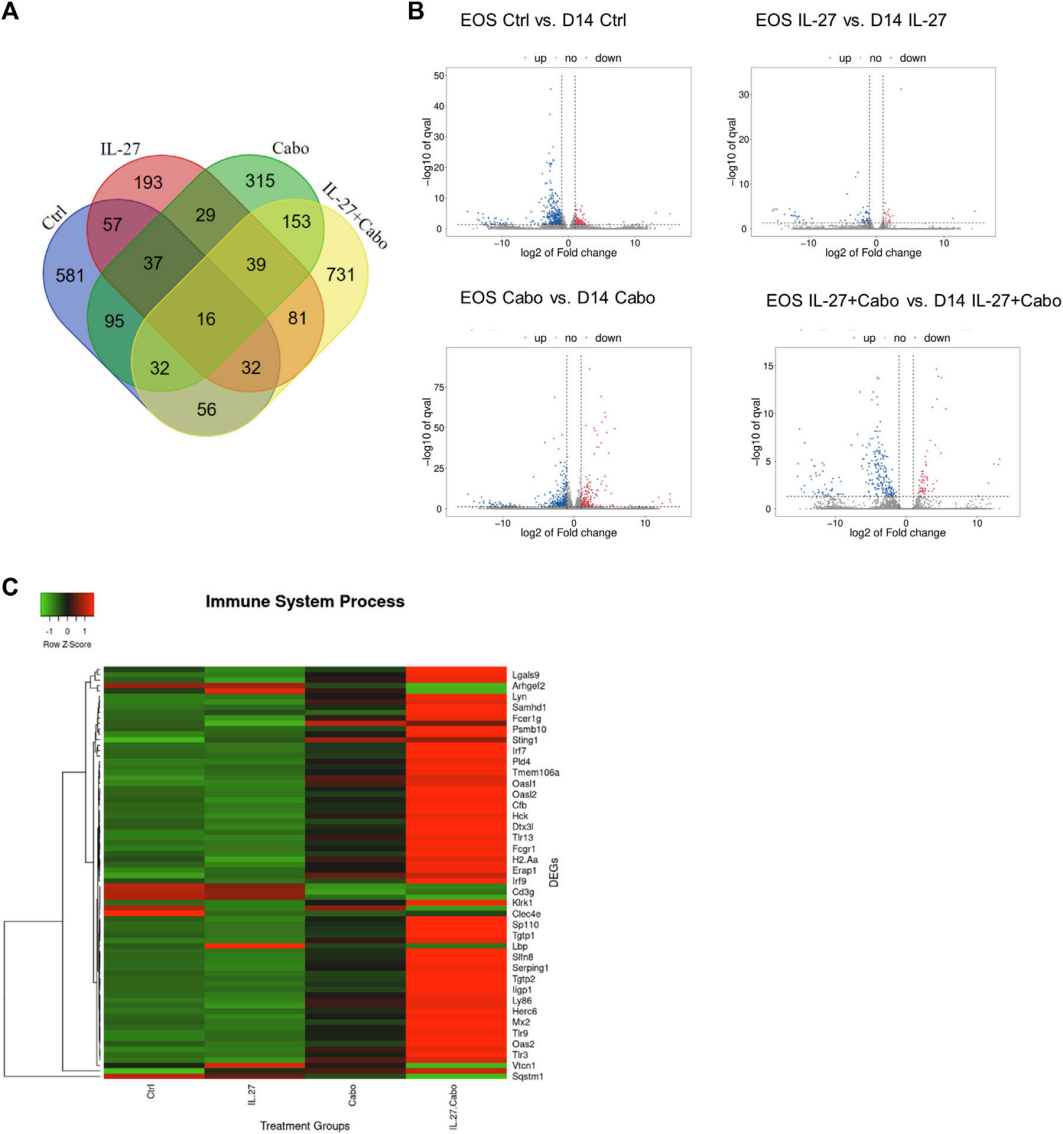
Comparison of DEGs in different groups on day 14 (D14) tumors relative to the tumors at the end-of-study (EOS): **(A)** Venn diagram and **(B)** volcano plots showing the DEGs in tumors collected 14 days post implantation compared to endpoint in different groups, **(C)** Heatmap showing the DEGs belonging to the GO group “Immune System Processes” compared among groups in the end-point tumors.

Chemotherapy resistance and tumor relapse remain major challenges in this field of research. An understanding of chemo-resistance pathways and identification of resistance biomarkers is necessary to inform treatment strategies and combinations for patients. Efforts have been made to identify chemotherapy resistance pathways in PCa including apoptotic pathways, heat-shock proteins, and inflammation and vasculature related pathways (Mahon et al., 2011). Hence, how IL-27 and cabo therapeutic mechanisms might have changed over time was examined. During the course of the *in vivo* experiment, cabo was administered for 14 days post tumor implantation. Some tumors (*n* = 3 mice) were harvested on day 14, when maximum therapeutic efficacy was observed in the treatment groups relative to control. The remaining animals remained in the study until they reached the ethical endpoint after which, the tumors were harvested, and the total RNA was sequenced. The cabo-treated groups (cabo and cabo + IL-27) had significantly higher survival compared to the control group. Thus, the DEGs in the end-of-study (EOS) tumors relative to the tumors at day 14 (D14) were examined to determine the impact of therapies in the anti-tumor response over time in different groups (Figure 9A, B).

Volcano plots showed that the gene expression remained the most constant between day 14 and EOS among all groups in the IL-27 treated tumors. This could be due to the sustained expression of IL-27, resulting in constant modulation of genes in the TME. Comparing the DEGs in the treatment groups using ANOVA (*p* < 0.05), the *Reactome 2022* database was used to reveal enriched pathways Supplementary Table 1. Many immune-related processes were enriched, indicating that the IL-27 and cabo therapy promoted long-lasting changes both in immune response and angiogenesis processes. Immune related genes (GO: *Immune System Process*) were upregulated in tumors treated with IL-27 and cabo (Figure 7C), demonstrating the sustained immunomodulatory effects of IL-27 and cabozantinib combination therapy.

## 4 Discussion

The current treatment strategies for patients with bone metastatic PCa are palliative and there is a need for treatments that not just reduce the tumor burden but also help reduce the damage caused to bone by metastatic lesions. In recent years, combinational treatment approaches have shown great promise in the field of cancer therapy. By using a combination of treatment strategies, it is possible to target tumors using complementary mechanisms, prevent resistance, and enhance the overall therapeutic efficacy. The use of chemotherapy in combination with immunotherapy is particularly promising to augment both agents’ anti-cancer potential. This work focuses on the investigation of a combination therapy comprising the cytokine IL-27 and cabo in a bone-metastatic PCa model.

The anti-tumor effects of IL-27 and cabo, along with their pro-osteogenic properties, make them promising candidates for PCa therapy. We hypothesized that the combination of IL-27 and cabo could enhance the immunogenicity of the tumor microenvironment, have potent anti-tumor effects, and improve bone quality. First, the impact of IL-27 gene therapy, delivered to tumor bearing mice, was determined by sequencing the total RNA isolated from the tumors. Analysis of DEGs revealed an upregulation of STAT1, STAT4, and CXCL11, indicating the activation of anti-tumor and immunomodulatory pathways. Additional GSEA analysis further identified enriched pathways associated with OC differentiation, immune-related processes, and pro-inflammatory responses. Of note, we also found some changes in gene expression with statistical significance in the group treated with cabo relative to the control group, including a 11.6-fold increase in the expression of the osteoblast marker IBSP relative to the control group.

The *in vivo* study evaluating the combination of IL-27 and cabo in an intratibial model showed interesting results. While IL-27 monotherapy did not show significant reduction in tumor size in this study, when combined with cabo, the anti-tumor effects were significantly enhanced. The anti-tumor effects of IL-27 gene therapy have been demonstrated before in subcutaneous tumor models (Di Carlo et al., 2014; Figueiredo et al., 2020), however, the more-clinically-relevant intratibial models tend to display far more aggressive growth in the bone (Dai et al., 2014) microenvironment in our laboratory’s experience, making this model much more challenging to treat. Animals treated with cabo or the combination also showed improved survival. The serum was analyzed for secreted Lucia activity to confirm successful gene delivery to skeletal muscle of the constructs bearing the reporter gene linked to the IL-27 transgene.

Further analysis of the molecular changes induced by IL-27 and cabo treatment revealed interesting insights into the immunomodulatory effects of the combination therapy. Tumors were harvested 14 days post implantation and RNA-sequencing was performed. The analysis of unique DEGs downregulated by IL-27+cabo treatment revealed the involvement of *T cell related processes* and the suppression of T_regs_. GSEA of the DEGs identified that IL-27 promotes NK cell activity while inhibiting B-cell activity. On the other hand, cabo was positively associated with *IFN response* and *chemokine production*. The IL-27+cabo group showed a combination of IL-27 and cabo’s effects on gene expression along with some additional synergistic effects. The enriched pathways in common between the monotherapies included *NK cell activation* and *type I IFN response*. Interferon signaling plays a vital role in PCa progression and metastasis (Owen et al., 2020). For instance, bone-metastatic progression of PCa has been linked to the loss of tumor‐intrinsic type I IFN loss, whereas restoring this signaling can lead to better outcomes. Further, IFNβ can also inhibit osteoclast differentiation (Zhang et al., 2012). Several muscle-related gene sets including *skeletal muscle tissue development*, *sarcomere organization*, and *skeletal muscle contraction* were also enriched in all samples. Similar muscle gene set enrichment has been observed in other studies characterizing PCa cells (Song et al., 2019; Sun et al., 2019). One possible explanation could be the presence of myofibroblasts or cancer-associated fibroblasts expressing muscle-related genes (Mayer and Leinwand, 1997). Additionally, the IL-27+cabo group was predicted to have improved T cell activation and reduced angiogenesis, providing further insights into the treatment’s mechanism of action.

The differences in immune infiltration among the treatment groups were estimated based on the DEGs obtained from RNA-sequencing, revealing that tumors treated with cabo exhibited a significant decrease in CD4^+^ cells and B-plasma cells compared to control tumors. Additionally, all treatment groups showed a predicted decrease in activated mast cell levels. Mast cells have been implicated in promoting the progression of prostate cancer by facilitating EMT. The suppression of mast cells by IL-27 and cabo treatment may have the potential to inhibit metastatic tumor growth. Consistent with the GSEA analysis, tumors treated with the combination of IL-27 and cabo showed a predicted downregulation of T_regs_ compared to monotherapy groups, suggesting a synergistic effect on immune-regulatory mechanisms. Moreover, the combination-treated tumors has an increased estimated infiltration of tumoricidal M1 macrophages and CD8^+^ T cells, indicating an effective anti-tumor response. The predicted decrease in endothelial cells/vessels in the treated tumors could be attributed to the anti-angiogenic effects of IL-27 and cabo. Overall, the IL-27+cabo combination therapy appeared to increase the infiltration of pro-inflammatory and anti-tumor immune effectors that could exert an anti-tumorigenic effect, decrease the presence of regulatory immune cells, and potentially inhibit angiogenesis within the tumor microenvironment.

In order to gain insights into the long-term therapeutic effects and changes in response over time, gene expression profiles of tumors harvested at the end-of-study (EOS) time point were compared to tumors harvested on day 14, when maximum therapeutic efficacy was observed. Volcano plots showed that the gene expression in IL-27 treated tumors changed the least among all groups, potentially due to sustained IL-27 expression leading to consistent modulation of genes in the tumor microenvironment. Comparison of DEGs (EOS) among the treatment groups using ANOVA analysis and enrichment analysis revealed several enriched immune-related processes, indicating that IL-27 and cabo therapy can induce long-lasting changes in immune response and angiogenesis. Immune-related genes were upregulated in tumors treated with IL-27 and cabo, demonstrating the sustained immunomodulatory effects of the combination therapy. Further optimization of the cabo dosage and duration can be performed to improve the survival bolstered by the sustained immune activity in the TME.

In the context of bone health, the combination therapy of IL-27 and cabo demonstrated positive effects on bone structure and architecture. µCT analysis and morphometry revealed that the combination therapy resulted in the healthiest bone relative to monotherapies, with improvements in trabecular architecture and bone-related parameters. The direct effects of IL-27 and cabo on bone cells were also explored, showing enhanced osteoblast differentiation and inhibition of osteoclast differentiation *in vitro*. These findings indicate the potential of IL-27 and cabo in not only reducing tumor burden but also in improving bone quality by modulating bone cell differentiation and factors affecting the bone-tumor interface, a crucial aspect in the management of bone-metastatic PCa. Overall, when comparing cabo to IL-27+cabo, the differences were not very pronounced. Cabo appears to exert a more dominant therapeutic effect in comparison to IL-27, although we have demonstrated some evidence of synergistic activity. We anticipate that as we work to enhance the vector delivery and expression of IL-27 in future studies, this synergistic effect will become more prominent.

In conclusion, this study provides valuable insights into the therapeutic potential of IL-27 and cabo combination therapy in the context of bone-metastatic PCa. The combination of IL-27 and cabo demonstrates enhanced anti-tumor effects, modulates the tumor microenvironment, and improves bone quality. This combinatorial approach holds promise in improving treatment outcomes and quality of life for patients. Overall, this research contributes to the development of innovative treatment strategies for bone metastatic PCa, addressing the critical unmet need in this patient population.

## 5 Conclusion

This investigation of the cytokine IL-27 and cabo, a small-molecule tyrosine kinase inhibitor, to treat BM-PCa highlights the potential of combination therapy in addressing the complex challenges associated with bone metastasis. Currently, no curative treatment exists for BM-PCa. This work demonstrates the synergistic effects of IL-27 and cabo in reducing the tumor burden, immuno-modulating the tumor microenvironment, and improving bone health.

Our experiments reveal that IL-27 and cabo therapy enhance the immunogenicity of the tumor microenvironment, promoting an anti-tumor immune response. The upregulation of immune-related genes and the activation of anti-tumor pathways underline the potential of this combinational approach in overcoming immune evasion and resistance mechanisms often observed in PCa. The therapeutic effects of IL-27 and cabo extend beyond tumor reduction, with significant improvements in bone quality. IL-27+cabo therapy results in better retention of bone structure and architecture *in vivo*. The combination was demonstrated to enhance OB differentiation and inhibit OC differentiation *in vitro*. Hence, the impact of the combination on bone health is due to its anti-tumor effects as well as direct pro-osteogenic effects. This result helps in addressing the research gap of a therapeutic strategy that can go beyond reducing the tumor burden by improving bone health.

The potential clinical implications of this combination therapy offer hope for improved outcomes and better quality of life for patients with bone metastatic PCa. Continued research in this field is essential to optimize treatment protocols, assess long-term efficacy, and ultimately translate these findings into clinical settings, addressing the urgent need for more effective and comprehensive treatments for patients with bone metastatic PCa.

## Data Availability

The data presented in the study are deposited in the NCBI GEO repository, accession number GSE241862.
