# Supplementary material for: Cabozantinib and IL-27 combinatorial therapy for bone-metastatic prostate cancer
Source: Front Mol Biosci. 2023 Sep 28;10:1259336. doi: 10.3389/fmolb.2023.1259336 (PMC10568464; doi:10.3389/fmolb.2023.1259336)
Supplement: Supplementary file 1 [file DataSheet1.docx]

Supplementary Material

Cabozantinib and IL-27 Combinatorial Therapy for Bone-Metastatic Prostate Cancer

Shreya Kumar^1^, Grace E. Mulia^1^, Marxa L. Figueiredo^1*^

^1^ Department of Basic Medical Sciences, College of Veterinary Medicine, Purdue University, West Lafayette, Indiana, 47907, USA

*** Correspondence:**Marxa L Figueiredo, Department of Basic Medical Sciences, College of Veterinary Medicine, Purdue University, 625 Harrison St, LYNN 2177, West Lafayette, IN 47904. Ph: 765-494-5790. Fax: 765-494-0781. Email: [mlfiguei@purdue.edu](mailto:mlfiguei@purdue.edu)

**
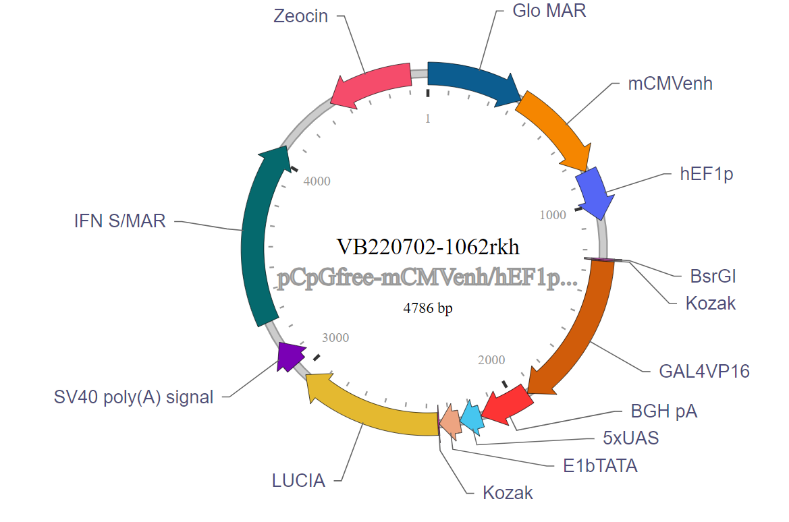
Supplementary Figure. 1**

**A**

**A**

**
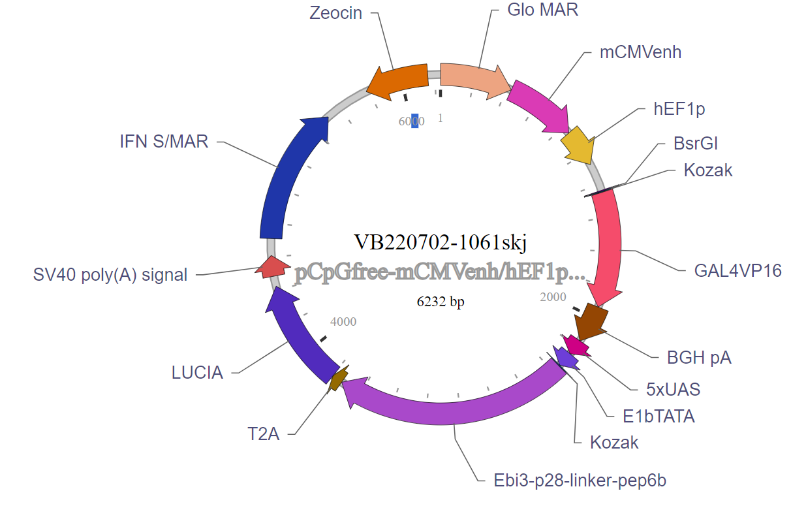
**

**B**

**A**

**Supplementary Figure 1.** **Construct maps of** (A) pTSTA-ctrl, and (B) pTSTA-IL27pepL vectors.

**Supplementary Figure. 2**

**B**

**B**

**A**

**A**


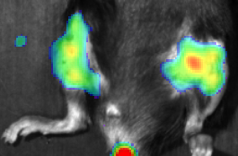

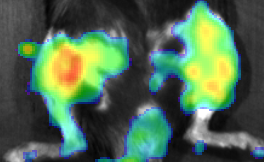

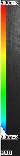

**Supplementary Figure 2.** **Luminescence assays to verify gene delivery and expression *in vivo*.** (A) BLI was used to detect luminescence in the muscle due to expression of the delivered plasmids containing a secreted luciferase, Lucia. Representative images and quantification using ROIs, (B) Luminescence detected in the serum of mice in relative light units (RLU), n=12-14, mean ± SEM, unpaired t-test, **p<0.01

**Supplementary Table. 1**

Reactome 2022 pathways enriched in DEGs (EOS tumors) among all the groups

| Term | P-value  (ANOVA p<0.05) |
| --- | --- |
| Immune System R-HSA-168256 | 2.21E-11 |
| Muscle Contraction R-HSA-397014 | 4.63E-09 |
| Striated Muscle Contraction R-HSA-390522 | 5.39E-09 |
| Extracellular Matrix Organization R-HSA-1474244 | 8.71E-09 |
| Interferon Alpha/Beta Signaling R-HSA-909733 | 9.57E-09 |
| Cytokine Signaling in Immune System R-HSA-1280215 | 1.82E-08 |
| Interferon Signaling R-HSA-913531 | 2.68E-08 |
| Hemostasis R-HSA-109582 | 6.35E-08 |
| Trafficking And Processing of Endosomal TLR R-HSA-1679131 | 2.14E-07 |
| Signal Transduction R-HSA-162582 | 8.31E-07 |
| Chemokine Receptors Bind Chemokines R-HSA-380108 | 1.23E-06 |
| Response Of EIF2AK1 (HRI) To Heme Deficiency R-HSA-9648895 | 1.26E-06 |
| Cell Surface Interactions at Vascular Wall R-HSA-202733 | 2.87E-06 |
| Post-translational Protein Phosphorylation R-HSA-8957275 | 2.24E-05 |
| Innate Immune System R-HSA-168249 | 2.50E-05 |
| Regulation Of IGF Transport and Uptake by IGFBPs R-HSA-381426 | 3.73E-05 |
| Interferon Gamma Signaling R-HSA-877300 | 3.73E-05 |
| Nervous System Development R-HSA-9675108 | 3.90E-05 |
| Integrin Cell Surface Interactions R-HSA-216083 | 6.21E-05 |
| Axon Guidance R-HSA-422475 | 6.89E-05 |
| Interleukin-10 Signaling R-HSA-6783783 | 1.27E-04 |
| Neutrophil Degranulation R-HSA-6798695 | 1.32E-04 |
| Platelet Activation, Signaling and Aggregation R-HSA-76002 | 2.19E-04 |
| Signaling By Interleukins R-HSA-449147 | 2.68E-04 |
